# Neuroprotective effects of D-Ala^2^GIP on Alzheimer's disease biomarkers in an APP/PS1 mouse model

**DOI:** 10.1186/alzrt174

**Published:** 2013-04-19

**Authors:** Emilie Faivre, Christian Hölscher

**Affiliations:** 1 School of Biomedical Sciences, Ulster University, Cromore road, Coleraine, BT52 1SA, UK

## Abstract

**Introduction:**

Type 2 diabetes mellitus has been identified as a risk factor for Alzheimer's disease (AD). An impairment of insulin signaling as well as a desensitization of its receptor has been found in AD brains. Glucose-dependent insulinotropic polypeptide (GIP) normalises insulin signaling by facilitating insulin release. GIP directly modulates neurotransmitter release, LTP formation, and protects synapses from the detrimental effects of beta-amyloid fragments on LTP formation, and cell proliferation of progenitor cells in the dentate gyrus. Here we investigate the potential therapeutic property of the new long lasting incretin hormone analogue D-Ala^2^GIP on key symptoms found in a mouse model of Alzheimer' disease (APPswe/PS1detaE9).

**Methods:**

D-Ala^2^GIP was injected for 21 days at 25 nmol/kg ip once daily in APP/PS1 male mice and wild type (WT) littermates aged 6 or 12 months of age. Amyloid plaque load, inflammation biomarkers, synaptic plasticity in the brain (LTP), and memory were measured.

**Results:**

D-Ala^2^GIP improved memory in WT mice and rescued the cognitive decline of 12 months old APP/PS1 mice in two different memory tasks. Furthermore, deterioration of synaptic function in the dentate gyrus and cortex was prevented in 12 months old APP/PS1 mice. D-Ala^2^GIP facilitated synaptic plasticity in APP/PS1 and WT mice and reduced the number of amyloid plaques in the cortex of D-Ala^2^GIP injected APP/PS1 mice. The inflammatory response in microglia was also reduced.

**Conclusion:**

The results demonstrate that D-Ala^2^GIP has neuroprotective properties on key hallmarks found in AD. This finding shows that novel GIP analogues have the potential as a novel therapeutic for AD.

## Introduction

Alzheimer's disease (AD), the most common type of dementia, is a devastating neurodegenerative disorder that has an increasingly high incidence in the elderly. At present, no treatment for AD is known. The disease is officially characterized by two principle hallmarks of pathology, which are amyloid plaques and neurofibrillary tangles (NFTs), composed of aggregated β-amyloid peptide and hyperphosphorylated tau respectively [1-5]. The most prominent feature of AD is the clinical decline in cognitive function, with an early impairment of episodic memory that later manifest as mild cognitive impairment and then later as AD dementia [6,7]. Other biomarkers include inflammation of the brain, loss of cholinergic neurons in the basal brain, glutamatergic neuronal loss, dendritic and synaptic loss among others [8,9].

Similarities between AD and Type 2 diabetes have been discovered in the last decades, and increased detailed knowledge of common physiological processes open up the opportunities for developing new treatments that may prevent or reduce the onset of AD [10,11]. Importantly, the disturbance in insulin signaling appears to be the main common impairment in both diseases [12-15]. Type 2 diabetes mellitus is characterized by resistance to insulin. A desensitisation of the insulin receptor has been also discovered in the brain of AD patients [13,16,17]. In addition, there is evidence to suggest that beta-amyloid oligomers bind to insulin receptors in the brain and cause a reduction of insulin receptor expression on dendrites [18,19]. This impairment in insulin signaling leads to an impairment of neuronal function, plaque formation and may lead to formation of NFTs [20-22]. This loss of insulin signaling in the brain may be one of the underlying mechanisms of neurodegeneration in AD. Insulin is a potent anabolic hormone, and activation of insulin receptor (IR) is essential for cell development, growth, and repair [23,24]. The IR is widely expressed in the brain [25] and its activation induces neuronal stem activation and dendritic sprouting [26,27]. Moreover, insulin plays a role in neuronal development, neuroprotection and memory [24,28]. Importantly, insulin can regulate levels of phosphorylated tau and is a potent neuroprotective factor, which can increase neuronal survival and protect neurons against the toxicity of amyloid fragments [29,30].

Since insulin signaling is desensitized in AD and since it is not sensible to give insulin administration to people as it would expedite the de-sensitisation in the long run, alternative strategies are investigated. Glucose-dependent insulinotropic polypeptide (GIP) is a potential candidate as it activates a parallel signaling pathway to insulin, namely, the incretin signaling pathway. GIP is an endogenous 42 amino acid peptide hormone, which is released by intestinal K-cells after a meal [31]. GIP analogues have been developed as potential treatments for type 2 diabetes, and ameliorate impaired insulin release from the pancreas and facilitate the normalisation of insulin signaling and hyperglycaemia [32,33]. The GIP receptor (GIPR) is expressed in various tissues and has been found in several brain regions with high levels of expression in the olfactory bulb and in the large pyramidal neurons in the hippocampus and cerebral cortex [34,35]. Activation of the GIPR leads to proliferation of neuronal progenitor cells and therefore may contribute to neurogenesis [35,36]. Moreover, GIP has neuroprotective and regenerative properties [37]. GIP prevents the detrimental effects of beta-amyloid on synaptic plasticity [38] and in spatial learning and memory during the water maze task [39]. Furthermore, GIP has been shown to promote axonal regeneration after sciatic nerve injury [40]. However, as GIP is quickly degraded by the enzyme dipeptidyl peptidase IV (DPP-IV) [41], several enzyme-resistant super-GIP molecules have been designed, such as the D-Ala^2^GIP [33,42]. Chronic injection of D-Ala^2^GIP for 21 days enhanced memory formation, synaptic neurotransmission (LTP) in the hippocampus, and progenitor cell proliferation in the dentate gyrus of wild type (WT) mice without inducing adverse side effects [43]. Additionally, a recent study has also shown protective effects of DAla^2^GIP on synaptic neurotransmission (LTP) and on object recognition memory when administrated twice daily over a 28-day period in mice on a high-fat diet [44]. Based on this information, we studied the effect of chronic systemic administration of D-Ala^2^GIP in an APP/PS1 mouse model of AD at different ages. Memory tasks and *in vivo *electrophysiology were used to evaluate potential effects of this GIP analogue on learning, memory and synaptic plasticity. Immunohistochemical staining was performed to assess the effects of D-Ala^2^GIP on cell proliferation, neurogenesis, synaptic density and on histological markers (amyloid plaques, dense-core plaques and neuroinflammation) of AD in APP/PS1 mice [45]. It is hoped that GIP analogues could prevent the detrimental effects of beta-amyloid on neuronal transmission and learning abilities at the very early stage of disease, and might reduce the number of plaques found in the brain in AD, and promote neuronal regeneration at later stages of the disease.

## Materials and methods

### Animals

Heterozygous male APPswe/PS1dE9 (APP/PS1) mice with a C57Bl/6J background were bought from Harlan (UK) and were bred at the University of Ulster with wild-type C57Bl/6J females also from Harlan (UK). The background and generation of APP/PS1 mice used in this study has been previously described in [46]. From the offspring, only male APP/PS1 mice, heterozygous for the APPswe/PS1dE9 transgenic construct, were used for the experiments, as well as age-matched non-AD mice, which are WT littermates, as control. Identification of the APP/PS1 transgenic and non-transgenic mice was performed according to the results of the PCR with primers specific for the APP sequence of the APP/PS1 construct, as outlined in [36]. Once mice were allocated to the APP/PS1 or control group, they were individually caged and received food and water *ad libitum*. Animals were maintained on a 12/12 light-dark cycle (lights on at 0800 h, off at 2000 h), in a temperature-controlled room (21.5°C ± 1). Every test was conducted during the light cycle. All experiments were licensed according to UK Home Office regulations (UK Animals Scientific Procedures Act 1986) and EU laws.

### Peptides

D-Ala^2^GIP was purchased from GL Biochem Ltd (Shanghai). The purity of the peptide was analysed by reversed-phase high performance liquid chromatography (HPLC) and characterised using matrix-assisted laser desorption/ionisation time of flight (MALDI-TOF) mass spectrometry.

### Drug treatment

**Figure 1:**
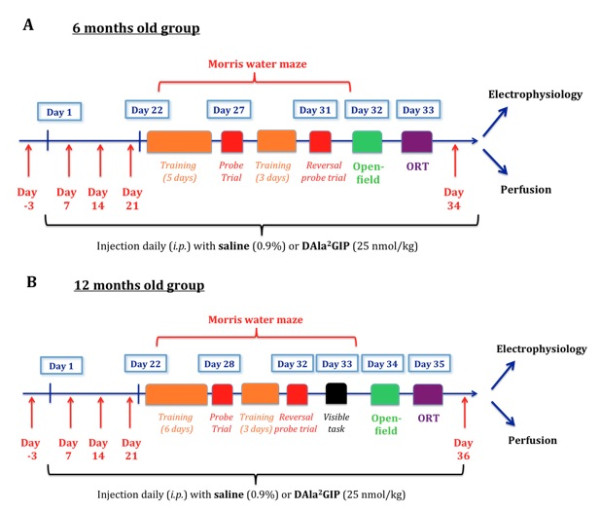
**Events sequence of the experimental procedure for (A) the 6-month-old groups, (B) the 12-month-old group**. Mice were injected for 21 days prior to different behavioural tasks, such as Morris water maze (MWM) task, open-field task and object recognition task (ORT). Injections were carried out until electrophysiological recordings or perfusions were performed. Arrows correspond to blood collection.

Animals were tested at two different ages, 6 months and 12 months. At these ages, APP/PS1 and WT mice were injected intraperitoneally (ip) at a volume of 10 ml/kg with saline solution (0.9% NaCl) or D-Ala^2^GIP at 25 nmol/kg body weight once daily for 21 days prior to the start of the behavioural study, and the injections were carried out until the end of the experimental study (see Figure 1). Thus, mice were allocated to four different treatment groups: saline-treated WT group, D-Ala^2^GIP-treated WT group, saline-treated APP/PS1 group and D-Ala^2^GIP-treated APP/PS1 group (see Table 1).

**Table 1:** Numbers of animals used for analysis of behavioural tasks and of electrophysiological recording of fEPSPs, depending on age, genotype and treatment given

Age	Genotype	Treatment	Behaviour	Electrophysiology
6 months	*WT*	Saline	***N *= 14**	***N *= 6**
6 months	*WT*	D-Ala^2^GIP	***N *= 14**	***N *= 6**
6 months	*APP/PS1*	Saline	***N *= 12**	***N *= 8**
6 months	*APP/PS1*	D-Ala^2^GIP	***N *= 12**	***N *= 8**
12 months	*WT*	Saline	***N *= 11**	***N *= 7**
12 months	*WT*	D-Ala^2^GIP	***N *= 12**	***N *= 7**
12 months	*APP/PS1*	Saline	***N *= 11**	***N *= 7**
12 months	*APP/PS1*	D-Ala^2^GIP	***N *= 11**	***N *= 6**

fEPSP, field excitatory post-synaptic potential; GIP, glucose-dependent insulinotropic polypeptide; N, number.

### Behavioural tasks

#### Object recognition task (ORT)

The apparatus consisted of an open-field arena (58 cm in diameter; 31 cm-high walls) constructed in aluminium with painted grey walls and grey floor. The open field was dimly illuminated by a 60-w lamp placed 2 m directly above the arena. The objects were red cubes (1.8 cm wide) and white balls (2.6 cm). The arena and objects were cleaned with 70% ethanol after each mouse trial to prevent the build-up of olfactory cues. Mice received a session of 5 minutes in the empty open field to habituate them to the apparatus and test room. The mouse was placed in the middle of the open field and was free to explore it. During that time, motor activity was recorded by total path, number of lines on the floor crossed and speed. The number of rearing events (forepaws elevated from the floor) was analysed as index of exploratory behaviour. The anxiety level was assessed by the percentage of time spent in the centre versus periphery of the arena and the number of grooming sessions. Grooming was defined as behaviour when the mouse stopped running and start licking, chewing and scratching at its fur. Twenty four hours after the habituation each mouse was subjected to a 10-minute acquisition trial, during which they were placed in the open field in the presence of object A, the first of two non-identical objects (a cube or a ball), situated 15 cm from the arena wall (acquisition task). On completion of 10 minutes exploration, the mouse was returned to its cage for a 3-h delay. After the retention interval, the mice were placed back into the box and exposed to the familiar object (A) and to a novel object (B) for a further 10 minutes (test task). The objects were placed in the same locations as the previous ones. The position of the novel object was fully counterbalanced (half left, half right) in a random manner to avoid preferences not based on novelty. Locomotor activity (number of lines crossed), speeds (cm/s), travel path and the total time spent exploring each of the two objects (when the animal's snout was directly toward the object at a distance ≤ 2 cm), were recorded. A recognition index was defined as the amount of time exploring the familiar object or the novel object over the total time spent exploring both objects, times 100, was used to measure recognition memory: (TA or TB/(TA + TB))*100. In the acquisition and retention trial, if the exploration time was < 30 s and < 15 s respectively, the mice were excluded from the trial. A video camera was fixed 2 m above the centre point of the arena and was attached to a video recorder, monitor and computer. The movement of the animals in the open field was tracked using a computerized tracking system (Biosignals, New York).

#### Morris water maze (MWM) task

The maze was made of white opaque plastic with a diameter of 120 cm and 40 cm-high walls, and was filled with water at 25°C to avoid hypothermia. A small escape platform (10 × 6.5 × 21.5 cm) was placed at a fixed position in the centre of one quadrant, 25 cm from the perimeter, and was hidden 1 cm beneath the water surface. The room contained a number of fixed visual cues on the walls. Four points equally spaced along the circumference of the pool (north, south, east, west) served as the starting position, which was randomised across the four trials each day. If an animal did not reach the platform within 90 s, it was guided to the platform where it had to remain for 30 s, before being returned to its home cage. The path length and escape latencies were recorded. One day after finishing the acquisition task, a probe trial was performed to assess the spatial memory (after a 24 h delay). The platform was removed from the maze and animals were allowed to swim freely for 60 s.

### Surgery and long-term potentiation recording in the hippocampus, area CA1

The technique used for testing long-term potentiation (LTP) in the hippocampus was exactly as previously described [46]. Mice were anaesthetised with urethane (ethyl carbamate, 1.8 g/kg, ip) for the duration of all experiments. Electrodes (tungsten with Teflon coating, Bilaney, UK) were implanted at the following coordinates: 1.5 mm posterior and 1.0 mm lateral for the recording electrode, and 2.0 mm posterior to bregma and 1.5 mm lateral to the midline for the stimulating electrode. The electrodes were lowered through the cortex and the upper layers of the hippocampus and into the CA1 region until the appearance of a negative deflecting excitatory post-synaptic potential (EPSP) that had a latency of *ca*.10 ms field (f)EPSPs were recorded on a computerised stimulating and recording unit (PowerLab, ADI instruments, USA). The program activated a constant current stimulus isolation unit (Neurolog, UK). Two different stimulation protocols were used: a weak high-frequency stimulation (HFS) protocol and a strong HFS protocol. Once fEPSP was found, different stimulation strengths were applied from 2.0 to 4.5 V in order to record later basic synaptic transmission. The stimulation strength, which triggered 50% of the maximum fEPSP response, was used for all further recordings for the weak HFS protocol, while the strong HFS protocol used a stimulation strength set at 75% of the maximum fEPSP response. This weak protocol was used in order to assess if peptides could facilitate LTP, as the control group was not potentiated at a maximal rate [38,47]. The stronger high frequency stimulation protocol consisted of three trains of 200 stimuli, inter-train interval 1 s, inter-stimulus interval 5 ms (200 Hz), with the stimulation strength set at 75% of the maximum fEPSP response. LTP was measured as percent of baseline fEPSP. This strong HFS protocol was found to potentiate LTP at a higher level under these stimulation conditions.

### Immunohistochemistry

For BrdU staining, mice received a single dose of BrdU (Sigma-Aldrich, St Louis, Mo, USA; Cat. No. B5002), 50 mg/kg of body weight at a concentration of 10 mg/ml. Ip injections were done 24 h before perfusion of the mouse brain.

After the LTP studies, animals were perfused transcardially with PBS followed by cold 4% paraformaldehyde in PBS. Brains were removed and fixed in 4% paraformaldehyde for at least 24 h before being transferred to 30% sucrose solution overnight. Brains were then snap-frozen using Envirofreez™ and coronal sections of 40-micron thickness were cut at a depth of -2 to -3 Bregma using a Leica cryostat. Sections were chosen according to stereological rules [48] with the first section taken at random and every fifth section afterwards. Between seven and thirteen sections were analysed per brain.

Immunohistochemistry was carried out for the ionized calcium binding adaptor molecule 1 (Iba1), a marker for activated microglia to measure the inflammation response [49], beta-amyloid plaques, congophilic plaques and doublecortin (DCX), a marker for immature neurons. All sections were incubated in 3% H_2_O_2 _to quench endogenous peroxidase activity. For Iba1 immunoreactivity, sections were incubated in 0.05 M Trisodium citrate (pH 9) at 90°C for 30 minutes to enhance antigen recognition. After blocking the sections in 5% normal serum to avoid non-specific antibody binding, they were incubated with rabbit polyclonal anti-Iba1 (1:2000, Wako, Germany, 016-20001) or goat polyclonal anti-DCX (1:200, Santa Cruz, CA, USA, sc-710), or polyclonal rabbit anti-synaptophysin primary antibody (Abcam, Cambridge, UK; Cat. No. ab7837), or a monoclonal mouse anti-BrdU antibody (Sigma-Aldrich, St Louis, Mo, USA; Cat. No. B2531), or rabbit polyclonal anti amyloid beta peptide (1:250, Invitrogen, UK, 71-5800) was added and incubated overnight at 4°C. For visualisation, Vectastain Elite and SG substrate (Vector laboratories, Burlingame, CA, USA) were used. Congo red staining for congophilic plaques was carried out as described by [50]. All staining was visualized by Axio Scope 1 (Zeiss, Germany) and analyzed using the multi threshold plug-in with Image J (NIH, USA). The background stain was subtracted in each histology analysis. As controls, sections without the primary antibody were used to evaluate the non-specific background stain.

### Determination of glucose levels

A few drops of blood were taken from the cut tip of the tail vein of mice. Blood glucose was measured instantly by an automated glucose oxidase procedure using the Ascencia^®^ Contour^®^ Blood Glucose Meter with corresponding analysis strips (Bayer Healthcare, Berkshire, UK).

### Determination of insulin levels by radioimmunoassay

Blood samples were collected from the cut tip of the tail vein of mice into microvette CB300 tubes (Sarstedt, Numbrecht, Germany; Cat. No. 16.446). Blood samples were then immediately centrifuged using a refrigerated centrifuge (Centrifuge 5415 R, Eppendorf AG, Hamburg, Germany) for 5 minutes at 12,000 × g. The supernatant plasma was aliquoted and stored at -80°C prior to insulin level determination.

Plasma insulin was assessed by radioimmunoassay (RIA). At least 24 h prior to use, a stock Dextran-coated charcoal (DCC) solution was also made up of 5% dextran T70-coated charcoal in stock RIA buffer. For each sample, 100 μl of guinea pig anti-porcine insulin antiserum and 100 μl of I^125 ^bovine insulin (10,000 cpm) were added. Samples were incubated with 1 ml of DCC for 20 minutes at 4°C, and then centrifuged at 900 × g for 20 minutes at 4°C. The resulting supernatant was gently removed and radioactivity in the charcoal, corresponding to unbound I^125 ^bovine insulin, was measured using an LKB Wallace gamma counter.

### Statistics

Statistical analyses were performed using Prism (Graphpad software Inc., USA) with the level of probability set at 95%. Standard error of the mean is shown in the figures. Data from the open field and immunostaining were analysed by one-way analysis of variance (ANOVA), followed by the Bonferroni post hoc test when the *P*-value was < 0.05, to measure the difference between groups. Student's paired *t-*test was used for the ORT and object location test (OLT) when the time spent exploring the familiar object/location was compared to the time spent exploring the novel object/location. Two-way repeated measures ANOVA was used to analyse differences between groups, effects over time and interactions for every behavioural measure (path length, escape latency and swim speed) of the acquisition task and to determine the difference in time spent between quadrants for each group in the probe trial, followed by the Bonferroni post hoc test. To evaluate difference in synaptic activity between groups, two-way repeated measures ANOVA was performed for the post-HFS baseline with group as independent variable and time as dependent variable. The same analysis was performed for the pre-HFS baseline to assess difference between groups over time.

## Results

### Body weights

#### Analysis at 6 months

**Figure 2:**
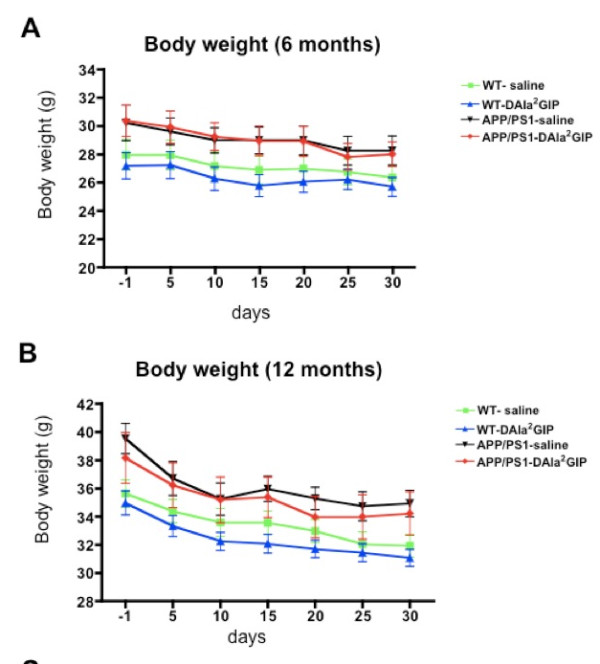
**Weight of APP/PS1 mice and their age-matched wild type control mice**. Mice were injected with saline and D-Ala2 glucose-dependent insulinotropic polypeptide (GIP) at 6 months of age. Mice were weighed one day before injections started and every 5 days over a period of 30 days. (**A**) Body weight at 6 months. Data represent mean ± standard error of the mean (SEM) of 14 wild type (WT) mice per group and 12 APP/PS1 mice per group (two-way analysis of variance, ANOVA). (**B**) Body weight at 12 months. Data represent mean ± SEM of 11 WT mice injected with saline, 12 WT mice injected with D-Ala^2^GIP and 11 APP/PS1 per group (two-way ANOVA).

On two-way ANOVA of mice at 6 months of age, there was an overall difference between groups (*F *= 14.20, *P *< 0.0001) but no difference over time (*F *= 2.07, *P *> 0.05), and there was no interaction of the two factors (*F *= 0.07, *P *> 0.05). Then, the body weight of WT mice injected with saline was compared with that of WT mice injected with D-Ala^2^GIP. On two-way ANOVA, there was no difference between groups (*F *= 2.77, *P *> 0.05), no effect over time (*F *= 0.91, *P *> 0.05) and no interaction of the two variables (*F *= 0.02, *P *> 0.05). The same analysis was performed comparing the body weight of APP/PS1 mice injected with saline and the body weight of APP/PS1 mice injected with D-Ala^2^GIP. On two-way ANOVA there was no difference between groups (*F *= 1.27, *P *> 0.05), no effect over time (F = 0.0004, *P *> 0.05) and no interaction of the two variables (*F *= 0.03, *P *> 0.05). However, there was a difference in body weight between WT and APP/PS1 mice that were both injected with saline (*F *= 12.47, *P *< 0.0001), as well as between WT and APP/PS1 mice that were both injected with D-Ala^2^GIP (*F *= 30.40, *P *< 0.0001). No difference was found over time in the saline groups (*F *= 0.82, *P *> 0.05) and D-Ala^2^GIP groups (*F *= 1.32, *P *> 0.05). No effect of interaction of both group and time was shown between the saline groups (*F *= 0.03, *P *> 0.05), or between the D-Ala^2^GIP groups (*F *= 0.19, *P *> 0.05). These statistics show that APP/PS1 mice had a higher body weight compared to WT control mice, which was not influenced by the saline or D-Ala^2^GIP injection (Figure 2A).

#### Analysis at 12 months

On two-way ANOVA of mice at 12 months of age there was an overall difference between groups (*F *= 16.85, *P *< 0.0001) and over time (*F *= 7.11, *P *< 0.0001), but no interaction of the two factors (*F *= 0.10, *P *> 0.05). Then, the body weight of WT mice injected with saline was compared with the body weight of WT mice injected with D-Ala^2^GIP. On two-way ANOVA there was a slight difference between groups (*F *= 6.25, *P *< 0.05), with an effect over time (*F *= 5.60, *P *< 0.0001), but there was no interaction of the two variables (*F *= 0.09, *P *> 0.05). The same analysis was performed comparing the body weight of APP/PS1 mice injected with saline and the body weight of APP/PS1 mice injected with D-Ala^2^GIP. There was no difference between groups (*F *= 1.08, *P *> 0.05), and no effect of interaction of the two variables (*F *= 0.06, *P *> 0.05), but a slight effect of time was found (*F *= 2.70, *P *< 0.05). On two-way ANOVA there was a difference in body weight between WT and APP/PS1 mice that were both mice injected with saline (*F *= 26.79, *P *< 0.0001) over time (*F *= 4.79, *P *< 0.0001), as well as between WT and APP/PS1 mice that were both injected with D-Ala^2^GIP (*F *= 21.08, *P *< 0.0001) with a slight effect of time (*F *= 2.88, *P *< 0.05). No effect of interaction of groups and time was shown between the saline groups (*F *= 0.27, *P *> 0.05) or between the D-Ala^2^GIP groups (*F *= 0.05, *P *> 0.05). These statistics show that APP/PS1 mice had a higher body weight compared to WT control mice, and the D-Ala^2^GIP peptide slightly decreased the body weight when injected in WT mice. Moreover a decrease in body weight was observed over time for all groups (Figure 2B).

### Blood glucose and plasma insulin levels

#### Analysis at 6 months

**Figure 3:**
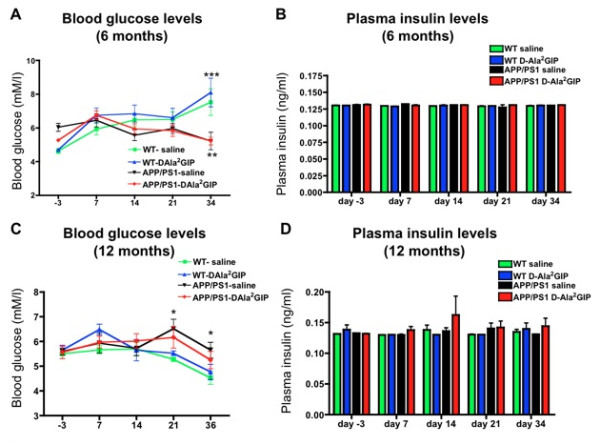
**Determination of (A) blood glucose levels, (B) plasma insulin levels in APP/PS1 mice and their age-matched wild type control mice, injected with saline or D-Ala2 glucose-dependent insulinotropic polypeptide (GIP) at 6 months old, and (C) blood glucose levels and (D) plasma insulin levels at 12 months**. Data represent mean ± standard error of the mean (SEM) of 14 wild type (WT) mice per group and 12 APP/PS1 mice per group (two-way analysis of variance, ANOVA, *P < 0.05; ** *P *< 0.01). (**C, D**) Data represent mean ± SEM of 11 WT mice injected with saline, 12 WT mice injected with D-Ala^2^GIP and 11 APP/PS1 per group (two-way ANOVA, *P < 0.05).

On two-way repeated measures ANOVA in mice at 6 months of age, there was a difference between groups in glucose concentrations (*F *= 4.08, *P *< 0.01), and over time (*F *= 7.23, *P *< 0.0001), and an influence of interaction between the two factors (*F *= 3.6, *P *< 0.0001) (Figure 3A). Bonferroni post hoc analysis revealed a significant increase in glucose concentrations in APP/PS1 mice injected with saline compared to WT mice injected with saline (*P *< 0.01) on day 34. Moreover, injection of D-Ala^2^GIP for 34 days increased blood glucose levels of APP/PS1 mice compared to WT mice (*P *< 0.0001). However, blood glucose levels stayed in a normal range (between 4 and 8 mM/l) for all groups over time. On two-way repeated measures ANOVA there was no difference between groups in plasma insulin levels (*F *= 1.75, *P *> 0.05), no difference over time (*F *= 1.91, *P *> 0.05), and no effect of interaction between the two factors (*F *= 0.96, *P *> 0.05) (Figure 3B).

#### Analysis at 12 months

On two way repeated measures ANOVA of mice at 12 months of age there was a slight difference between groups in glucose concentrations (*F *= 3.84, *P *< 0.05), and a difference over time (*F *= 7.14, *P *< 0.0001), which was not influenced by interaction between the two factors (*F *= 1.39, *P *> 0.05) (Figure 3C). Bonferroni post hoc analyses revealed a significant decrease of glucose concentrations in APP/PS1 mice injected with saline compared to WT mice injected with saline (*P *< 0.05) on day 21 and 36. However, blood glucose levels stayed in the normal range (between 4 and 8 mM/l) for all groups over time. On two-way repeated measures ANOVA there was no difference between groups in plasma insulin levels (*F *= 1.56, *P *> 0.05), no difference over time (*F *= 0.74, *P *> 0.05) and no effect of interaction for the two factors (*F *= 0.54, *P*p > 0.05) (Figure 3D).

### Effect of D-Ala^2^GIP treatment on spontaneous behaviour of APP/PS1 mice and WT littermates

#### Analysis at 6 months

In the open-field task at 6 months of age, the spontaneous behaviour of WT and APP/PS1 mice injected with saline or D-Ala^2^GIP was similar. On one-way ANOVA there was no difference between groups in path length (*F *= 1.99, *P *> 0.05), number of lines crossed (*F *= 1.59, *P *> 0.05), speed (*F *= 1.99, *P *> 0.05), exploration levels (*F *= 0.29, *P *> 0.05), grooming events (*F *= 0.85, *P *> 0.05), or the ratio of time spent in the centre of the arena to the periphery (*F *= 1.29, *P *> 0.05) (data not shown).

#### Analysis at 12 months

In the open-field task at 12 months of age, the spontaneous behaviour of WT and APP/PS1 mice injected with saline or D-Ala^2^GIP was similar. On one-way ANOVA there was no difference between groups in path length (*F *= 1.14, *P *> 0.05), number of lines crossed (*F *= 0.94, *P *> 0.05), speed (*F *= 1.14, *P *> 0.05), exploration levels (*F *= 0.56, *P *> 0.05, grooming events (*F *= 0.21, *P *> 0.05), and the ratio of time spend in the centre of the arena to the periphery (*F *= 0.07, *P *> 0.05) (data not shown).

### Effect of D-Ala^2^GIP treatment on object recognition memory of APP/PS1 mice and WT littermates

#### Analysis at 6 months

**Figure 4:**
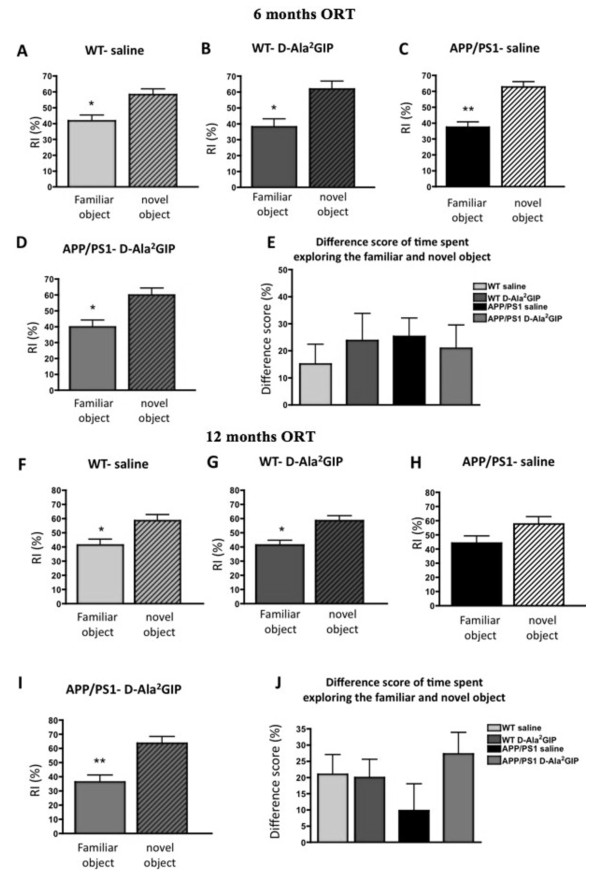
**Assessment of recognition memory of 6-month-old APP/PS1 mice and wild type age-matched mice injected with saline and with D-Ala2 glucose-dependent insulinotropic polypeptide (GIP) for 32 days and assessment of recognition memory of 12-month-old mice injected for 34 days**. Recognition index (RI) for familiar and novel locations during the test task (after a 3-h delay). Data represent mean ± standard error of the mean (SEM) of wild type (WT) mice injected with (**A**) saline (*n *= 14) and (**B**) D-Ala^2^GIP (*n *= 14), and APP/PS1 mice injected with (**C**) saline (*n *= 12) and (**D**) D-Ala^2^GIP (*n *= 12), (Student's paired *t-*test, *P < 0.05; **P < 0.01). (**E**) Difference in score of time spent exploring familiar and novel objects for each group (one-way analysis of variance, ANOVA). At 12 months, WT mice injected with (**F**) saline (*n *= 11) and (**G**) D-Ala^2^GIP (*n *= 11), and APP/PS1 mice injected with (**H**) saline (*n *= 11) and (**I**) D-Ala^2^GIP (*n *= 11) (Student's paired *t-*test, *P < 0.05; **P < 0.01). (**J**) Difference in score for time spent exploring familiar and novel objects for each group (one-way ANOVA).

In the test trial, there was a difference (Student's paired *t*-test) in the recognition index (RI) of novel vs familiar objects for WT mice injected with saline (*t *= 2.28, *P *< 0.05) and with D-Ala^2^GIP (25 nmol/kg) (*t *= 2.37, *P *< 0.05), as well as for APP/PS1 mice injected with saline (t = 3.71, *P *< 0.01) and with D-Ala^2^GIP (*t *= 2.28, *P *< 0.05). On one-way ANOVA there was no difference between groups in the difference in score between the time spent exploring the novel and the familiar object (*F *= 0.27, *P *> 0.05). These results indicate that all groups had an intact object recognition memory at 6 months of age (Figure 4).

#### Analysis at 12 months

In the test trial, there was a difference (Student's paired *t*-test) in the RI of novel vs familiar objects for 12-month-old WT mice injected with saline (*t *= 2.07, *P *< 0.05) and with D-Ala^2^GIP (25 nmol/kg) (*t *= 2.51, *P *< 0.05), showing an intact object recognition memory in these groups. APP/PS1 mice injected with D-Ala^2^GIP spent significantly more time exploring the novel object than the familiar one (*t *= 2.80, *P *< 0.01), while APP/PS1 control mice injected with saline did not discriminate between the familiar and the novel task, as no difference was found in the time spent between them (*t *= 1.33, *P *> 0.05), reflecting an impairment in recognition memory for this group. On one-way ANOVA, there was no difference between groups in the difference in score between the time exploring the novel and the familiar object (*F *= 1.15, *P *> 0.05); even the APP/PS1 group injected with saline solution showed a trend towards reduction of the difference in score in comparison to the other groups but this difference was not significant (Figure 4).

### Effect of D-Ala^2^GIP treatment on spatial learning and memory of APP/PS1 mice and WT littermates

#### Analysis at 6 months

During the acquisition task, all mice learned to locate the hidden escape platform. On two-way repeated measures ANOVA there was a decrease in escape latency across trials of training in the acquisition trial (trials: *F *= 11.47, *P *< 0.0001), but there was no difference between groups (*F *= 0.64, *P *> 0.05) and no interaction of training trials and groups (*F *= 0.86, *P *> 0.05). A decrease in path length was also found across training trials (*F *= 13.75, *P *< 0.0001), although no differences were detected between groups (*F *= 0.75, *P *> 0.05). There was no interaction of training trials and groups (*F *= 0.97, *P *> 0.05). On two-way repeated measures ANOVA there was a difference in swim speed over time (trials: *F *= 7.01, *P *< 0.0001) but not between groups (*F *= 1.96, *P *> 0.05). No effect was found for the interaction of both factors (*F *= 1.19, *P *> 0.05) (data not shown).

In the probe trial, on one-way ANOVA there was an effect of quadrant preference for WT mice injected with saline (F = 9.70, *P *< 0.0001), and D-Ala^2^GIP (*F *= 7.60, *P *< 0.0001), and the APP/PS1 mice injected with saline (*F *= 11.83, *P *< 0.0001) and D-Ala^2^GIP (*F *= 6.47, *P *< 0.01), indicating that all groups remembered the location of the escape platform. In the WT saline-injected group, Bonferroni post hoc analysis showed increased time of stay in the target quadrant (south-west, S-W) compared to the south-east (S-E) (*P *< 0.001), north-east (N-E) (*P *< 0.001) and north-west (N-W) (*P *< 0.01) quadrants. In the WT D-Ala^2^GIP-injected group, Bonferroni post hoc analysis showed increased time of stay in the target quadrant (S-W) compared to the S-E (*P *< 0.01), N-E (*P *< 0.01) and N-W (*P *< 0.001) quadrants. In the APP/PS1 saline-injected group, Bonferroni post hoc analyses showed increased time of stay in the target quadrant (S-W) compared to the S-E (*P *< 0.05), N-E (*P *< 0.001) and N-W (*P *< 0.05) quadrants. APP/PS1 mice injected with D-Ala^2^GIP spent more time in the target S-W quadrant compared to the other (*P *< 0.01) (data not shown).

The probe trial was further analysed using one-way ANOVA, to analyse the difference in time spent in the target quadrant between all groups. No significant difference was found between groups for this parameter (*F *= 0.37, *P *> 0.05). Moreover, there was no difference between groups in the time spent crossing the exact previous location of the platform (*F *= 0.50, *P *> 0.05).

During the reversal task, all mice learned the new location of the platform. On two-way repeated measures ANOVA there was a significant decrease in escape latency across trials of training in the acquisition trial (trials: *F *= 5.76, *P *< 0.0001). A difference in escape latency was also found between groups (*F *= 4.97, *P *< 0.01), which was not influenced by interaction of training trials and groups (*F *= 0.83, *P *> 0.05). Then, on two-way repeated measures ANOVA to compare the escape latency between WT injected with saline and those injected with D-Ala^2^GIP there was a difference over time (trials: *F *= 3.17, *P *< 0.0001), but not between groups (*F *= 2.44, *P *> 0.05), and there was no effect of interaction between both factors (*F *= 0.54, *P *> 0.05). The same analysis comparing the APP/PS1 group injected with saline and that injected with D-Ala^2^GIP showed a significant difference between groups in escape latency (*F *= 4.36, *P *< 0.05) and over time (*F *= 4.00, *P *< 0.0001), which was not influenced by interaction between factors (*F *= 0.82, *P *> 0.05). On two-way ANOVA there was a difference in escape latency between WT and APP/PS1 mice that were both injected with saline (*F *= 4.85, *P *< 0.05), as well as between WT and APP/PS1 mice that were both injected with D-Ala^2^GIP (*F *= 3.91, *P *< 0.05). A difference was also found over time for the saline (*F *= 2.73, *P *< 0.01) and D-Ala^2^GIP groups (*F *= 3.66, *P *< 0.0001). No effect of interaction of both group and time was shown between the saline groups (*F *= 1.18, *P *> 0.05), or between the D-Ala^2^GIP groups (*F *= 0.76, *P *> 0.05).

On two-way repeated measures ANOVA there was a significant decrease in path length across trials of training in the acquisition trial (trials: *F *= 4.28, *P *< 0.0001). A difference in escape latency was also found between groups (*F *= 6.54, *P *< 0.0001), which was not influenced by interaction of training trials and groups (F = 0.64, *P *> 0.05). Then, on two-way repeated measures ANOVA to compare the escape latency between WT injected with saline and D-Ala^2^GIP, there was a difference over time (trials: *F *= 16.06, *P *< 0.0001), but not between groups (*F *= 2.58, *P *> 0.05) and no effect of interaction between the two factors (*F *= 3.47, *P *> 0.05). The same analysis was conducted to compare the APP/PS1 group injected with saline and that injected with D-Ala^2^GIP, and this showed a significant difference in the escape latency training trials (*F *= 1.86, *P *< 0.05), but not between groups (*F *= 0.63, *P *> 0.05) and no effect of interaction between factors (*F *= 0.43, *P *> 0.05). On two-way ANOVA there was no difference in escape latency between WT and APP/PS1 mice that were both injected with saline (*F *= 9.7, *P *> 0.05), or between WT and APP/PS1 mice that were both injected with D-Ala^2^GIP (*F *= 8.74, *P *> 0.05). No difference was found over time for the saline groups (*F *= 1.93, *P *> 0.05) and the D-Ala^2^GIP groups (*F *= 2.80, *P *> 0.05). No effect of interaction of group and time was shown between the saline groups (*F *= 0.95, *P *> 0.05), or between the D-Ala^2^GIP groups (*F *= 0.65, *P *> 0.05). On two-way repeated measures ANOVA there was no difference between groups in swim speed over time, or interaction between the two factors (trials: *F *= 1.41, *P *> 0.05; groups: *F *= 0.61, *P *> 0.05; interaction: *F *= 1.07, *P *> 0.05).

In the reversal probe trial, on one-way ANOVA there was an effect of quadrant preference for WT mice injected with saline (*F *= 7.67, *P *< 0.0001), and D-Ala^2^GIP (*F *= 5.13, *P *< 0.01), and the APP/PS1 mice injected with saline (*F *= 4.76, *P *< 0.01) and D-Ala^2^GIP (F = 7.45, *P *< 0.0001), indicating that all groups remembered the location of the escape platform. In the WT saline-injected group, Bonferroni post hoc analysis showed increased time of stay in the target quadrant (N-W) compared to the N-E (*P *< 0.05) and S-E (*P *< 0.001) quadrants. In the WT D-Ala^2^GIP-injected group, Bonferroni post hoc analyses showed increased time of stay in the target quadrant (N-W) compared to the N-E (*P *< 0.01) and S-E (*P *< 0.05) quadrants. In the APP/PS1 saline-injected group, Bonferroni post hoc analysis showed increased time of stay in the target quadrant (N-W) compared to the others (*P *< 0.05). APP/PS1 mice injected with D-Ala^2^GIP spent more time in the target S-W quadrant than in the N-E (*P *< 0.05) and S-E (*P *< 0.001) and the S-W (*P *< 0.05) quadrants.

The reversal probe trial was further analysed using one-way ANOVA, to analyse the difference between all groups in the time spent in the target quadrant. No significant difference was found between groups for this parameter (*F *= 0.48, *P *> 0.05). Additionally, one-way ANOVA did not show any difference in the spent time crossing the exact previous location of the platform between groups (*F *= 0.83, *P *> 0.05).

#### Analysis at 12 months

During the acquisition task, on two-way repeated measures ANOVA there was a decrease in escape latency across trials of training in the acquisition trial (trials: *F *= 10.20, *P *< 0.0001), with a difference between groups (*F *= 5.92, *P *< 0.0001), which was not influenced by interaction of training trials and groups (*F *= 0.67, *P *> 0.05). Then, on two-way repeated measures ANOVA to compare the escape latency between WT injected with saline D-Ala^2^GIP we found a difference over time (trials: *F *= 4.20, *P *< 0.0001), and between groups (*F *= 9.29, *P *> 0.005), but no effect of interaction between the two factors (*F *= 0.79, *P *> 0.05). The same analysis between the APP/PS1 group injected with saline or with D-Ala^2^GIP and showed a significant difference in escape latency between groups (*F *= 6.15, *P *< 0.05) and over time (*F *= 6.86, *P *< 0.0001), which was not influenced by interaction between factors (*F *= 0.0.48, *P *> 0.05). On two-way ANOVA, there was no difference in escape latency between WT and APP/PS1 mice that were both injected with saline (*F *= 0.42, *P *> 0.05), as well as between WT and APP/PS1 mice both injected with D-Ala^2^GIP (*F *= 1.77, *P *> 0.05). However, a difference was found over time for the saline groups (*F *= 3.35, *P *< 0.0001) and D-Ala^2^GIP groups (*F *= 8.14, *P *< 0.0001). No effect of interaction of group and time was shown between the saline groups (*F *= 0.69, *P *> 0.05), or between the D-Ala^2^GIP groups (*F *= 0.45, *P *> 0.05).

A decrease in path length was also found across training trials (*F *= 17.60, *P *< 0.0001), and differences were detected between groups (*F *= 6.043, *P *< 0.0001). There was no interaction of training trials and groups (*F *= 0.73, *P *> 0.05). Then, on two-way repeated measures ANOVA to compare the path length between WT injected with saline or D-Ala^2^GIP there was a difference over time (trials: *F *= 8.48, *P *< 0.0001), but not between groups (*F *= 0.01, *P *< 0.05), and no effect of interaction between the two factors was detected (*F *= 0.96, *P *> 0.05). The same analysis between the APP/PS1 group injected with saline or D-Ala^2^GIP showed a significant difference in escape latency over trials (*F *= 9.60, *P *< 0.0001), but not between groups (*F *= 61.36, *P *> 0.05), and there was no effect of interaction between factors (*F *= 0.47, *P *> 0.05). On two-way ANOVA there was a difference in escape latency between WT and APP/PS1 mice that were both mice injected with saline (*F *= 4.99, *P *< 0.05), as well as between WT and APP/PS1 mice both injected with D-Ala^2^GIP (*F *= 1.77, *P *> 0.05). A difference was found over time for the saline groups (*F *= 9.12, *P *< 0.0001) and D-Ala^2^GIP groups (*F *= 9.28, *P *< 0.0001). No effect of interaction of group and time was shown between the saline groups (*F *= 0.88, *P *> 0.05), or between the D-Ala^2^GIP groups (*F *= 0.60, *P *> 0.05). On two-way repeated measures ANOVA there was a difference in swim speed over time (trials: *F *= 7.49, *P *< 0.0001) but not between groups (*F *= 2.30, *P *> 0.05). No effect was found for the interaction of the two factors (*F *= 0.55, *P *> 0.05) (data not shown).

**Figure 5:**
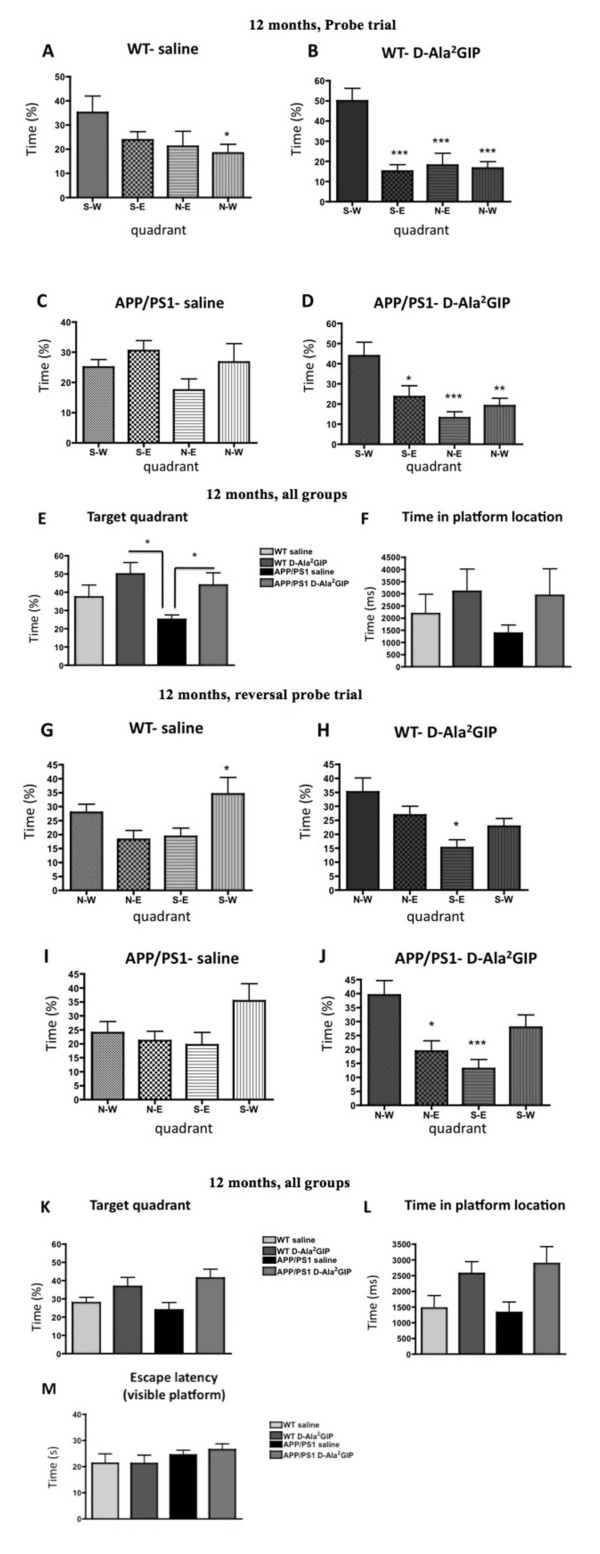
**Evaluation of spatial memory during the probe trial of 12-month-old APP/PS1 mice and wild type age-matched mice injected with saline or with D-Ala2 glucose-dependent insulinotropic polypeptide (GIP) for 27 days and reversal probe trial of 12-month-old mice injected for 31 days**. Time spent in each quadrant of the water maze during the probe trial for wild type (WT) mice injected with (**A**) saline and with (**B**) D-Ala^2^GIP and APP/PS1 mice injected with (**C**) saline and with (**D**) D-Ala^2^GIP. Data represent mean ± standard error of the mean (SEM) of 11 WT mice injected with saline, 12 WT mice injected with D-Ala^2^GIP and 11 APP/PS1 per group. (**E**) Time spent in the target quadrant for all groups. (**F**) Time spent crossing the platform previously located during acquisition task. Data represent mean ± SEM of 11 WT mice injected with saline, 12 WT mice injected with D-Ala^2^GIP and 11 APP/PS1 per group (one-way analysis of variance, ANOVA). At 12 months, time spent in each quadrant of the water maze during the probe trial for WT injected with (**G**) saline and with (**H**) D-Ala^2^GIP and APP/PS1 mice injected with (**I**) saline and with (**J**) D-Ala^2^GIP. Data represent mean ± SEM of 11 WT mice injected with saline, 12 WT mice injected with D-Ala^2^GIP and 11 APP/PS1 per group (one-way ANOVA; post hoc test, *P < 0.05; **P < 0.01; ***P < 0.001). (**K**) Time spent in the target quadrant for all groups. (**L**) Time spent crossing the platform previously located during acquisition task (one-way ANOVA, * *P *< 0.05). (**M**) Evaluation of visual acuity of 12-month-old APP/PS1 mice and WT age-matched mice injected with saline or with D-Ala^2^GIP, during the visible platform task. Escape latency for the four trials was average for each mouse (one-way ANOVA).

In the probe trial, one-way ANOVA showed an effect of quadrant preference for WT mice injected with saline (*F *= 3.02, *P *< 0.05) or D-Ala^2^GIP (*F *= 12.06, *P *< 0.0001), and the APP/PS1 mice injected with D-Ala^2^GIP (*F *= 7.47, *P *< 0.0001). However, APP/PS1 mice injected with saline spent an equal amount of time in each quadrant of the pool (*F *= 0.16, *P *> 0.05), indicating that this group did not remember the location of the escape platform. In the WT saline-injected group, Bonferroni post hoc analysis showed increased time of stay in the target quadrant (S-W) compared to the N-W (*P *< 0.05) quadrant. In the WT D-Ala^2^GIP-injected group, Bonferroni post hoc analyses showed increased time of stay in the target quadrant (S-W) compared to the other quadrants (*P *< 0.001). In the APP/PS1 D-Ala^2^GIP-injected group, Bonferroni post hoc analyses showed increased time of stay in the target quadrant (S-W) compared to the S-E (*P *< 0.05), N-E (*P *< 0.001) and N-W (*P *< 0.01) quadrants. See Figure 5.

The probe trial was further analysed using one-way ANOVA of the difference between all groups in the time spent in the target quadrant (Figure 5). A significant difference between groups was found for this parameter (*F *= 3.31, *P *< 0.05). Bonferroni post hoc analysis showed a decrease in time spent in the target quadrant for the APP/PS1 group injected with saline compared to the WT group injected with D-Ala^2^GIP (*P *< 0.05). Moreover, on one-way ANOVA there was no difference in the time spent crossing the exact previous location of the platform between groups (*F *= 0.87, *P *> 0.05). However, the APP/PS1 control group showed a trend towards a reduction in the time spent in the platform location compared to the other groups.

During the reversal task, on two-way repeated measures ANOVA there was a significant decrease in escape latency across trials of training in the acquisition trial (trials: *F *= 4.39, *P *< 0.0001). A difference in escape latency was also found between groups (*F *= 7.69, *P *< 0.0001), which was not influenced by interaction of training trials and groups (*F *= 0.72, *P *> 0.05). Then, on two-way repeated measures ANOVA to compare the escape latency between WT injected with saline or D-Ala^2^GIP, there was a difference over time (trials: *F *= 3.00, *P *< 0.0001), and between groups (*F *= 19.83, *P *< 0.0001), which was not influenced by interaction between factors (*F *= 0.45, *P *> 0.05). The same analysis was conducted between the APP/PS1 group injected with saline or D-Ala^2^GIP and showed a significant difference in escape latency between trials (*F *= 1.19, *P *< 0.05), but not between groups (*F *= 3.21, *P *> 0.05), and no effect of interaction between both factors was detected (*F *= 1.28, *P *> 0.05). On two-way ANOVA there was no difference in escape latency between WT and APP/PS1 mice that were both injected with saline (*F *= 1.45, *P *< 0.05), or over time (*F *= 1.55, *P *> 0.05). Moreover, on two-way ANOVA there was no difference in escape latency between WT and APP/PS1 mice that were both injected with D-Ala^2^GIP (*F *= 1.56, *P *> 0.05), but a difference was found over time (*F *= 4.87, *P *< 0.0001). No effect of interaction of group and time was shown between the saline groups (*F *= 0.40, *P *> 0.05), or between the D-Ala^2^GIP groups (*F *= 0.45, *P *> 0.05).

On two-way repeated measures ANOVA there was a significant decrease in path length across trials of training in the acquisition trial (trials: *F *= 2.79, *P *< 0.01), but not between groups (*F *= 0.99, *P *> 0.05), and no effect of interaction of both factors was detected (*F *= 1.13, *P *> 0.05). On two-way repeated measures ANOVA there was no difference between groups in swim speed over time, or interaction between the two factors (trials: *F *= 0.46, *P *> 0.05; groups: *F *= 0.84, *P *> 0.05; interaction: *F *= 0.91, *P *> 0.05) (data not shown).

In the reversal probe trial, one-way ANOVA showed an effect of quadrant preference for WT mice injected with saline (*F *= 3.80, *P *< 0.05), or D-Ala^2^GIP (*F *= 5.41, *P *< 0.01), and the APP/PS1 mice injected with D-Ala^2^GIP (*F *= 7.39, *P *< 0.0001). However, APP/PS1 mice injected with saline spent an equal amount of time in each quadrant of the pool (*F *= 02.40, *P *> 0.05), indicating that this group did not remember the location of the escape platform. In the WT saline-injected group, Bonferroni post hoc analyses showed increased time of stay in the S-W quadrant compared to the N-E (*P *< 0.05) quadrant, indicating that this group did not remember the location of the escape platform. In the WT D-Ala^2^GIP-injected group, Bonferroni post hoc analyses showed increased time of stay in the target quadrant (N-W) compared to the S-E (*P *< 0.05) quadrant. The APP/PS1 D-Ala^2^GIP-injected with D-Ala^2^GIP spent more time in the target N-W quadrant compared to the N-E (*P *< 0.05) and S-E (*P *< 0.001) quadrants. See Figure 5b.

**Figure 6:**
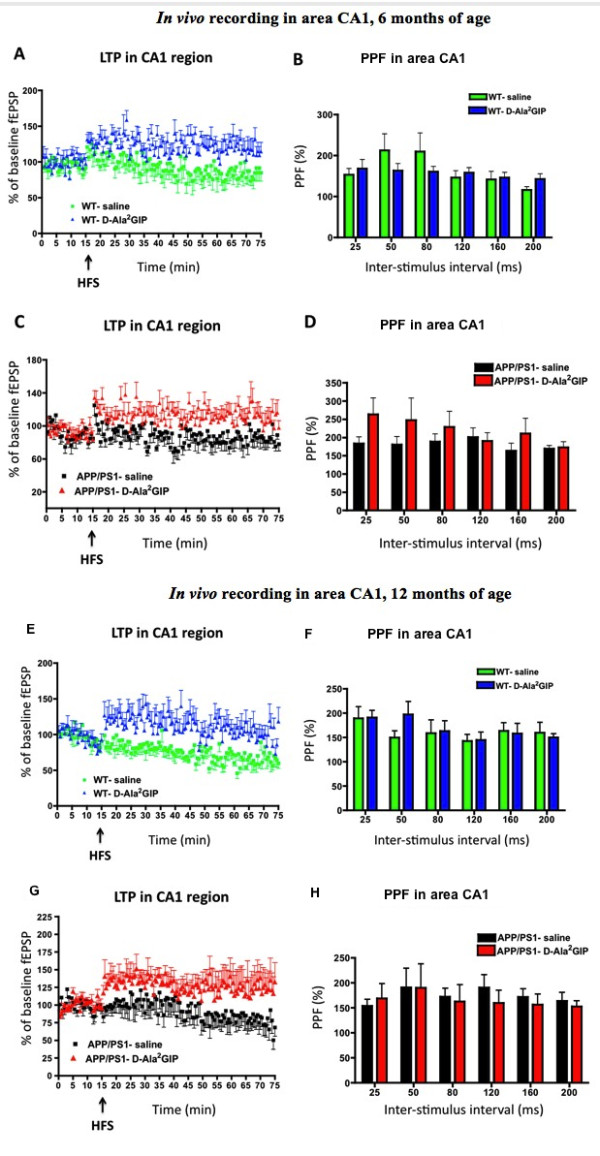
***In vivo *recording of field excitatory postsynaptic potentials (fEPSPs), from the stratum radiatum in response to stimulation of the Schaffer collateral/commissural pathway in wild type mice, injected with saline and D-Ala2 glucose-dependent insulinotropic polypeptide (GIP and in APP/PS1 mice**. At 6 months, (**A**) potentiation of the fEPSP after high-frequency stimulation and (**B**) paired-pulse facilitation in area CA1. The inter-stimulus interval was set at 25 to 200 ms. Data represent mean ± standard error of the mean (SEM) of six mice per group (two-way repeated measures analysis of variance, ANOVA). In the APP/PS1 mice, (**C**) potentiation of the fEPSP after high-frequency stimulation and (**D**) paired-pulse facilitation in area CA1. The inter-stimulus interval was set at 25 to 200 ms. Data represent mean ± SEM of eight mice per group (two-way repeated measures ANOVA). At 12 months, (**E**) potentiation of the fEPSP after high frequency stimulation and (**F**) paired-pulse facilitation in area CA1. The inter-stimulus interval was set at 25 to 200 ms. Data represent mean ± SEM of seven mice per group (two-way repeated measures ANOVA). In the APP/PS1 mice, (**G**) potentiation of the fEPSP after high-frequency stimulation and (**H**) paired-pulse facilitation in area CA1. The inter-stimulus interval was set at 25 to 200 ms. Data represent mean ± SEM of seven mice for the saline group and six for the D-Ala^2^GIP group (two-way repeated measures ANOVA).

The reversal probe trial was further analysed using a one-way ANOVA of the difference between all groups in the time spent in the target quadrant (Figure 6). A significant difference between groups was found for this parameter (*F *= 3.27, *P *< 0.05). Moreover, one-way ANOVA showed a difference in the time spent crossing the exact previous location of the platform between groups (*F *= 3.42, *P *< 0.05). Both the WTand APP/PS1group injected with saline showed a trend towards a reduction in the time spent in the platform location compared to the other groups, but the difference was not significant. In the visible platform task, one-way ANOVA did not show any difference in escape latency between groups in visual acuity (*F *= 0.89, *P *> 0.05) (Figure 5b).

In summary, the drug treatment did not have any effects in mice that were 6 months old, at an age when the amyloid plaque load and the associated learning impairments had not yet developed. However, D-Ala^2^GIP improved memory in WT mice and rescued the cognitive decline of 12 month-old APP/PS1 mice in two different memory tasks.

### Effect of D-Ala^2^GIP treatment on synaptic plasticity of APP/PS1 mice and WT littermates

#### Analysis at 6 months

The pre-HFS baselines of WT mice at 6 months of age were analysed by two-way repeated measures ANOVA (Figure 6a). No difference was found between the saline group and D-Ala^2^GIP group (*F *= 1.24, *P *> 0.05), and no effect of time (*F *= 0.74, *P *> 0.05) or interactive effect of group and time was determined (*F *= 0.61, *P *> 0.05), indicating that pre-HFF baselines were stable and similar between groups over time. On two-way repeated measures ANOVA there was a clear difference in post-HFS baselines between the WT group injected with saline and the WT group injected with D-Ala^2^GIP (groups: *F *= 289.3, *P *< 0.0001), but not over time (*F *= 0.63, *P *> 0.05) and no interactive effect of group and time was found (interaction: *F *= 0.93, *P *> 0.05). Altogether, these results indicate that HFS stimulation induced robust LTP in the WT D-Ala^2^GIP-injected mice compared to the control group. On two-way repeated measures ANOVA there was no difference between groups in paired-pulse facilitation (PPF) (*F *= 0.26, *P *> 0.05). Inter-stimulus delay (*F *= 0.57, *P *> 0.05) and interaction between both factors (*F *= 1.11, *P *> 0.05) were not significant.

The pre-HFS baselines of APP/PS1 mice at 6 months of age were analysed by two-way repeated measures ANOVA. No difference was found between the saline and D-Ala^2^GIP group (*F *= 0.00007, *P *> 0.05), and no effect of time (*F *= 0.94, *P *> 0.05) and no interactive effect of group and time was determined (*F *= 0.60, *P *> 0.05), indicating that pre-HFF baselines were stable and similar between groups over time. On two-way repeated measures ANOVA there was a clear difference of post-HFS baselines between the APP/PS1 group injected with saline and the APP/PS1 group injected with D-Ala^2^GIP (groups: *F *= 309.2, *P *< 0.0001), but not over time (*F *= 0.94, *P *> 0.05) and no interactive effect of group and time was found (interaction: *F *= 0.60, *P *> 0.05). Altogether, these results indicate that HFS stimulation induced robust LTP in the APP/PS1 D-Ala^2^GIP-injected mice compared to the control group. On two-way repeated measures ANOVA there was a difference between groups in PPF (*F *= 4.07, *P *< 0.05). Inter-stimulus delay (*F *= 0.69, *P *> 0.05) and interaction between the two factors (*F *= 0.58, *P *> 0.05) were not significant. See Figure 6a.

#### Analysis at 12 months

The pre-HFS baselines of WT mice at 12 months of age were analysed by two-way repeated measures ANOVA (Figure 6b). No difference was found between the saline and D-Ala^2^GIP group (*F *= 1.18, *P *> 0.05), but an effect of time was determined (*F *= 2.00, *P *< 0.01), which was not influenced by interaction of group and time (*F *= 0.57, *P *< 0.01), indicating that pre-HFF baselines were similar between groups. On two-way repeated measures ANOVA there was a clear difference of post-HFS baselines between the WT group injected with saline and the WT group injected with D-Ala^2^GIP (groups: *F *= 427.0, *P *< 0.0001), but not over time (*F *= 1.19, *P *> 0.05) and no interactive effect of group and time was found (interaction: *F *= 0.46, *P *> 0.05). Altogether these results indicate that HFS stimulation induced robust LTP in the WT D-Ala^2^GIP-injected mice compared to the control group. On two-way repeated measures ANOVA there was no difference between groups on PPF (*F *= 0.28, *P *> 0.05). Inter-stimulus delay (*F *= 1.41, *P *> 0.05) and interaction between both factors (*F *= 0.58, *P *> 0.05) were not significant.

The pre-HFS baselines of APP/PS1 mice at 12 months of age were analysed by two-way repeated measures ANOVA. No difference was found between the saline and the D-Ala^2^GIP group (*F *= 0.00001, *P *> 0.05), and no effect of time (*F *= 1.18, *P *> 0.05) and no interactive effect of group and time was determined (*F *= 1.18, *P *> 0.05), indicating that pre-HFF baselines were stable and similar between groups over time. On two-way repeated measures ANOVA there was a clear difference of post-HFS baselines between the APP/PS1 group injected with saline and the APP/PS1 group injected with D-Ala^2^GIP (groups: *F *= 234.1.2, *P *< 0.0001), but not over time (*F *= 0.54, *P *> 0.05) and no interactive effect of group and time was found (interaction: *F *= 0.26, *P *> 0.05). Altogether these results indicate that HFS stimulation induced robust LTP in the APP/PS1 D-Ala^2^GIP-injected mice compared to the control group. On two-way repeated measures ANOVA there was no difference between groups on PPF (*F *= 0.33, *P *> 0.05). Inter-stimulus delay (*F *= 0.39, *P *> 0.05) and interaction between the two factors (*F *= 0.16, *P *> 0.05) were not significant. See Figure 6b.

In conclusion, D-Ala^2^GIP facilitated synaptic plasticity in APP/PS1 and WT mice at all ages tested.

### Effect of D-Ala^2^GIP treatment on cell proliferation and neurogenesis of APP/PS1 mice and WT littermates

#### Analysis at 6 months

**Figure 7:**
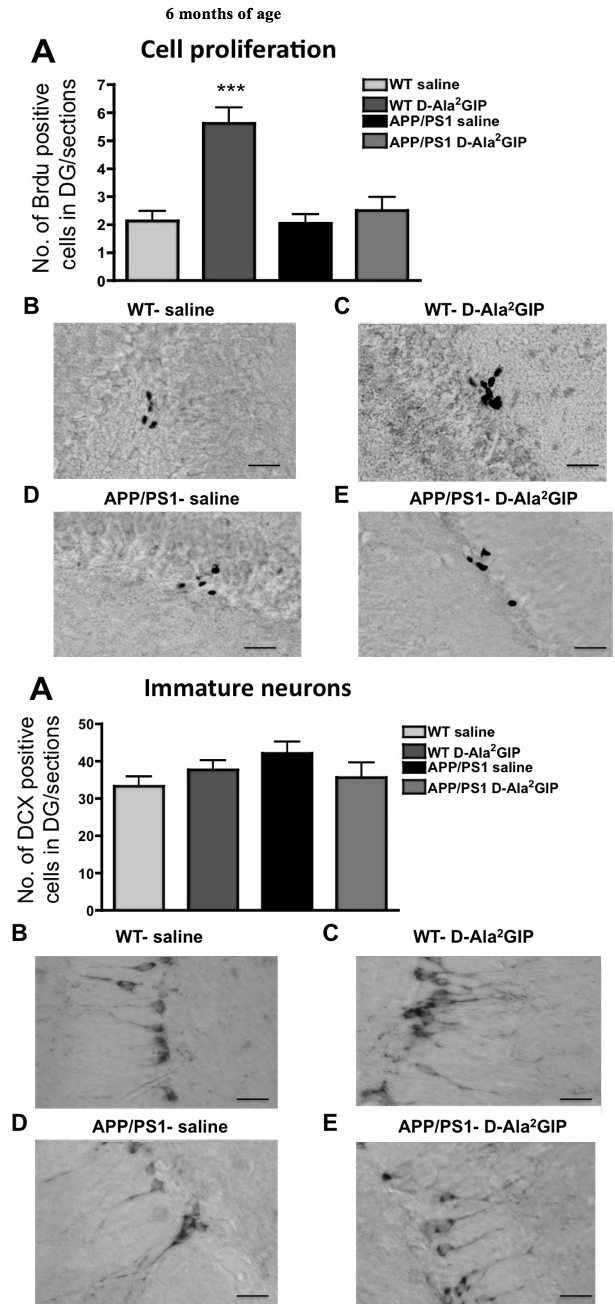
**Histological analysis of biomarkers in the brain**. Top half of Figure: effect of D-Ala2 glucose-dependent insulinotropic polypeptide (GIP) treatment on cell proliferation of 6-month-old APP/PS1 mice and wild type (WT) littermates. Quantification of (**A**) BrdU-positive cells in the dentate gyrus (DG0 of APP/PS1 and WT mice. Data show mean ± standard error of the mean (SEM) of six mice per group (one-way analysis of variance, ANOVA, ***P < 0.001). The micrographs show examples of the DG of WT mice injected with (**B**) saline and (**C**) D-Ala^2^GIP, and APP/PS1 mice injected with (**D**) saline and (**E**) D-Ala^2^GIP peptide, immunohistologically stained against BrdU (scale bar: 210 μm). Bottom half of Figure: effect of D-Ala^2^GIP treatment on neurogenesis of 6-month-old APP/PS1 mice and WT littermates. Quantification of (**A**) DCX-positive cells in the DG of APP/PS1 and WT mice. Data show mean ± SEM of six mice per group (one-way ANOVA). The micrographs illustrate examples of the DG of WT mice injected with (**B**) saline and (**C**) D-Ala^2^GIP, and APP/PS1 mice injected with (**D**) saline and (**E**) D-Ala^2^GIP peptide, immunohistologically stained against DCX (scale bar: 210 μm).

On one-way ANOVA there was a difference between groups in BrdU-positive cells in the dentate gyrus (DG) (*F *= 12.90, *P *< 0.0001, Figure 7a). The Bonferroni post hoc test showed that the DG of WT mice injected with D-Ala^2^GIP contained significantly more BrdU-positive cells than the other groups (*P *< 0.001). However, on one-way ANOVA there was no difference between groups in the number of double-cortin-positive young neurons (*F *= 1.49, *P *> 0.05) (Figure 7b).

#### Analysis at 12 months

**Figure 8:**
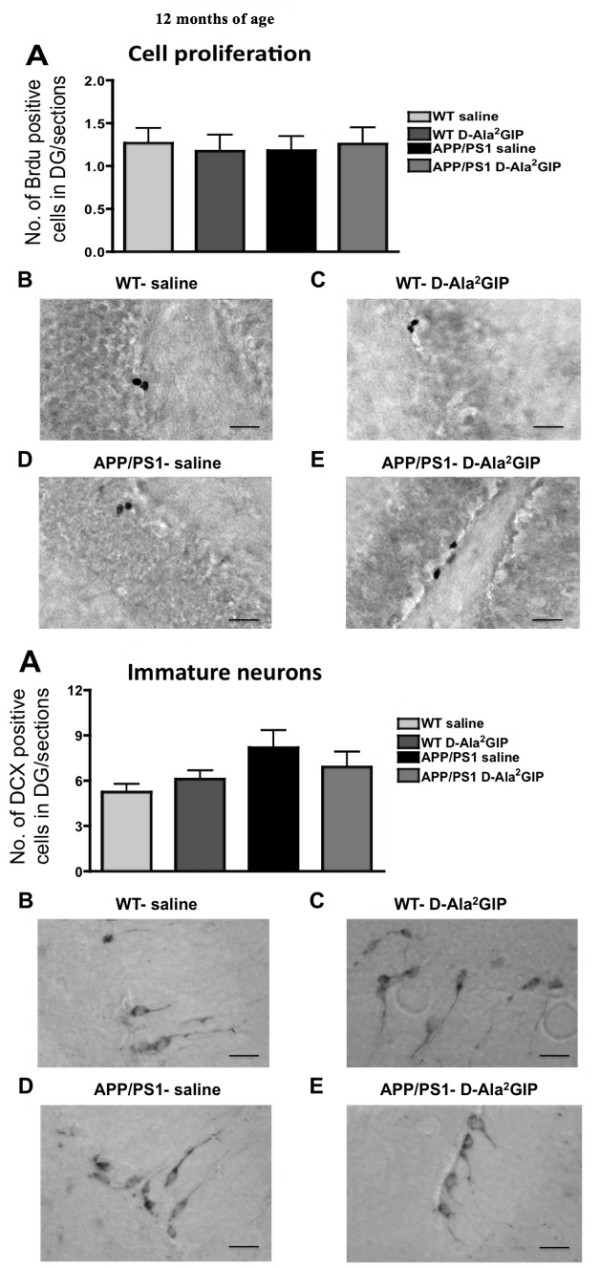
**Histological analysis of biomarkers in the brain**. Top half of Figure: effect of D-Ala2 glucose-dependent insulinotropic polypeptide (GIP) treatment on cell proliferation of 12-month-old APP/PS1 mice and wild type (WT) littermates. Quantification of (**A**) BrdU-positive cells in the dentate gyrus (DG) of APP/PS1 and WT mice. Data show mean ± standard error of the mean (SEM) of six mice per group (one-way analysis of variance, ANOVA). The micrographs illustrate examples of the DG of WT mice injected with (**B**) saline and (**C**) D-Ala^2^GIP, and APP/PS1 mice injected with (**D**) saline and (**E**) D-Ala^2^GIP peptide, immunohistologically stained against BrdU (scale bar: 210 μm). Bottom half of Figure: effect of D-Ala^2^GIP treatment on neurogenesis of 12-month-old APP/PS1 mice and WT littermates. Quantification of (**A**) doublecortin (DCX)-positive cells in the DG of APP/PS1 and WT mice. Data show mean ± SEM of 6 mice per group (one-way ANOVA). The micrographs illustrate examples of the DG of WT mice injected with (**B**) saline and (**C**) D-Ala^2^GIP, and APP/PS1 mice injected with (**D**) saline and (**E**) D-Ala^2^GIP peptide, immunohistologically stained against DCX (scale bar: 210 μm).

On one-way ANOVA there was no difference between groups in BrdU-positive cells in the DG at 12 months of age (*F *= 0.07, *P *> 0.05, Figure 8a). Moreover, there was no difference between groups in the number of double-cortin-positive young neurons (*F *= 2.11, *P *> 0.05) (Figure 8b).

### Effect of D-Ala^2^GIP treatment on synaptic density of APP/PS1 mice and WT littermates

#### Analysis at 6 months

**Figure 9:**
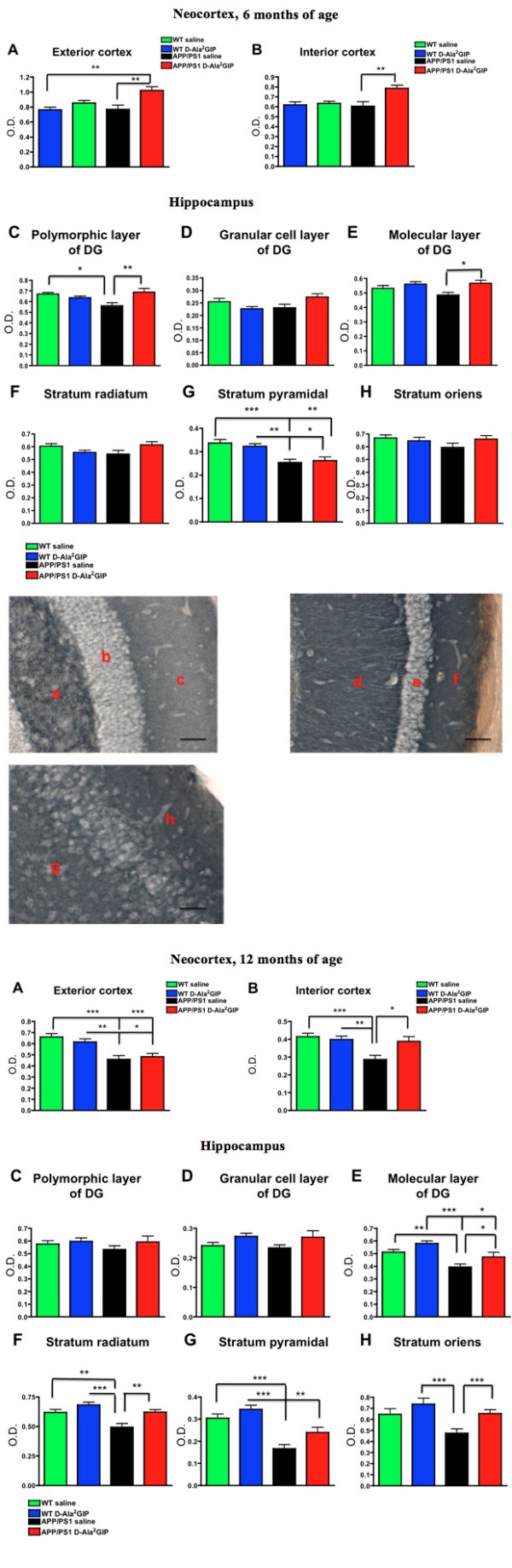
**Histological analysis of biomarkers in the brain**. Top half of Figure: effect of D-Ala2 glucose-dependent insulinotropic polypeptide (GIP) treatment on synaptic density on 6-month-old APP/PS1 mice and wild type (WT) age-matched littermates. Quantification of levels of expression of synaptophysin in (**A**) the exterior neocortex (layers 1 to 3), and (**B**) the interior neocortex (layers 4 to 6). Data show mean ± standard error of the mean (SEM) of six mice per group (one-way analysis of variance, ANOVA, **P < 0.01). Quantification of levels of expression of synaptophysin in (**C**) the polymorphic layer of the dentate gyrus (DG), (**D**) the granular cell layer of the DG, (**E**) the molecular layer of the DG, (**F**) the stratum radiatum, (**G**) the stratum pyramidal and (**H**) the stratum oriens. Data show mean ± SEM of six mice per group (one-way ANOVA, * *P *< 0.05; ** *P *< 0.01; *** *P *< 0.001). Representative images of synaptophysin stained-brains of WT mice (scale bar: 440 μm). The photographs on the top row show the polymorphic layer of the DG (**a**), the granular cell layer of the DG (**b**), the molecular layer of the DG **(c)**, the stratum radiatum (**d**), the stratum pyramidal (**e**) and the stratum oriens (**f**). On the micrograph, interior neocortex (**g**) and exterior neocortex (**h**) stained against synaptophysin is shown. Bottom half of Figure: effect of D-Ala^2^GIP treatment on synaptic density on 12-month-old APP/PS1 mice and WT age-matched littermates. Quantification of levels of expression of synaptophysin in (**A**) the exterior cortex, and (**B**) the interior cortex. Data show mean ± SEM of six mice per group (one-way ANOVA). Hippocampus: (**C**) the polymorphic layer of the DG, (**D**) the granular cell layer of the DG, (**E**) the molecular layer of the DG, (**F**) the stratum radiatum, (**G**) the stratum pyramidal and (**H**) the stratum oriens. Data show mean ± SEM of six mice per group (one-way ANOVA). * *P *< 0.05; **P < 0.01; ***P < 0.001.

On one-way ANOVA there was a difference between groups in levels of expression of synaptophysin in the exterior cortex (layers 4 to 6) (*F *= 6.62, *P *< 0.0001) (Figure 9a) and interior cortex (layers 1 to 3) (*F *= 5.72, *P *< 0.01). The Bonferonni post hoc test showed that D-Ala^2^GIP treatment increased levels of synaptophysin in the interior cortex in APP/PS1 mice compared with the saline-treated APP/PS1 (*P *< 0.01), and in the exterior cortex compared with both saline-treated APP/PS1 and WT mice (*P *< 0.01).

On one-way ANOVA there was a difference between groups in levels of expression of synaptophysin in the polymorphic layer (hilus) of the DG (*F *= 4.95, *P *< 0.01), the molecular layer of the DG (*F *= 3.55, *P *< 0.05) and the stratum pyramidale (CA1) (*F *= 9.14, *P *< 0.0001). A decrease in synaptophysin levels was found in APP/PS1 mice compared to the WT control group in the polymorphic layer (hilus) (P < 0.05), and a decrease was found in the stratum pyramidale in the saline group (*P *< 0.001) and in the D-Ala^2^GIP group (*P *< 0.05). D-Ala^2^GIP treatment increased levels of synaptophysin in APP/PS1 mice compared with the saline-treated APP/PS1 mice in the polymorphic layer of the DG (*P *< 0.01), as well as in the molecular layer of the DG (*P *< 0.05). However, D-Ala^2^GIP treatment did not change synaptophysin expression in WT and APP/PS1 mice in the granular cell layer of the DG (*F *= 2.65, *P *> 0.05), in the stratum radiatum (*F *= 2.15, *P *> 0.05), or the stratum oriens (*F *= 1.24, *P *> 0.05). See Figure 9a.

#### Analysis at 12 months

On one-way ANOVA there was a difference between groups in levels of expression of synaptophysin in the exterior cortex (layers 406) (*F *= 10.52, *P *< 0.0001) and interior cortex (layers 1 to 3) (*F *= 6.84, *P *< 0.0001). The Bonferonni post hoc test showed a decrease of synaptophysin expression in the 12 month-old APP/PS1 mice compared to their respective WT control in the exterior cortex (*P *< 0.001 and *P *< 0.05). In the interior cortex, APP/PS1 mice treated with saline exhibited lower levels of synaptophysin expression than the WT group (*P *< 0.001 compared to the saline group, and *P *< 0.01 compared to the D-Ala^2^GIP group), while D-Ala^2^GIP treatment increased levels of synaptophysin expression in APP/PS1 mice compared with the saline-treated APP/PS1 (*P *< 0.05).

On one-way ANOVA there was a difference between groups in levels of expression of synaptophysin in the molecular layer of the DG (*F *= 8.71, *P *< 0.0001), the stratum radiatum (*F *= 9.39, *P *< 0.0001), the stratum pyramidale (*F *= 15.45, *P *< 0.0001), and the stratum oriens (*F *= 5.88, *P *< 0.01) in area CA1. A decrease in synaptophysin levels was found in saline-treated APP/PS1 mice compared to the saline-treated WT control group in the molecular layer and the stratum radiatum (*P *< 0.01) and the stratum pyramidal (*P *< 0.001). Moreover, a decrease of synaptophysin levels was also found in D-Ala^2^GIP-treated APP/PS1 mice compared to the D-Ala^2^GIP-treated WT control group in the stratum pyramidal (*P *< 0.01). However, D-Ala^2^GIP treatment increased levels of synaptophysin in APP/PS1 mice in the molecular layer of the DG (*P *< 0.05), in the stratum radiatum and stratum pyramidal (*P *< 0.01), as well as in the stratum oriens (*P *< 0.001). No change was detected in the polymorphic layer (hilus) (*F *= 0.76, *P *> 0.05) and in the granular cell layer of the DG (*F *= 1.97 *P *> 0.05) of WT and APP/PS1 mice. See Figure 9b.

### Effect of D-Ala^2^GIP treatment on plaque formation and inflammation in the cortex of APP/PS1 mice

#### Analysis at 6 months

**Figure 10:**
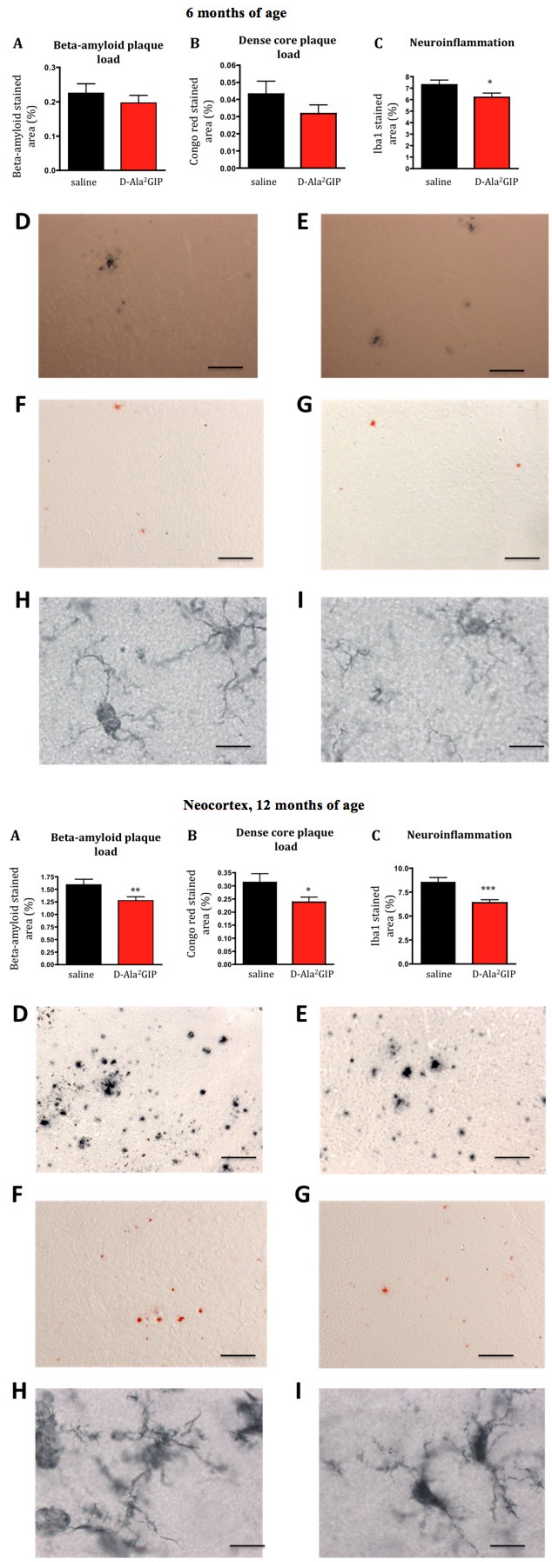
**Histological analysis of biomarkers in the brain**. Top half of figure: effect of D-Ala2 glucose-dependent insulinotropic polypeptide (GIP) treatment on three hallmarks of Alzheimer's disease in 6-month-old APP/PS1 mice. (**A**) Quantification of beta-amyloid plaque, (**B**) of dense core plaque load in the cortex of APP/PS1 and, (**C**) of activated microglia (neuroinflammation) in the cortex of APP/PS1 mice. The percentage of stained cortex area was used as measurement for these three hallmarks. Data show mean ± standard error of the mean (SEM) of six mice per group (Student's unpaired *t-*test, *P < 0.05). Micrographs: The figures on the top row illustrate examples of the cortex of APP/PS1 mice injected with (**D**) saline and (**E**) D-Ala^2^GIP, immunohistologically stained against β-amyloid (scale bar: 880 μm). The figures in the middle row illustrate example of cortex of APP/PS1 mice injected with (**F**) saline and (**G**) D-Ala^2^GIP, stained with Congo red (scale bar: 880 μm). The figures on the bottom row illustrate examples of cortex of APP/PS1 mice injected with (**H**) saline and (**I**) D-Ala^2^GIP, immunohistologically stained against Iba1 (scale bar: 80 μm). Bottom half of Figure: effect of D-Ala^2^GIP treatment on three hallmarks of Alzheimer's disease in 12-month-old APP/PS1 mice. (**A**) Quantification of beta-amyloid plaque, (**B**) of dense core plaque load in the cortex of APP/PS1 and, (**C**) of activated microglia (neuroinflammation) in the cortex of APP/PS1 mice. The percentage of stained cortex area was used as measurement for these three hallmarks. Data show mean ± SEM of six mice per group (Student's unpaired *t-*test, *P < 0.05; **P < 0.01; ***P < 0.001). Micrographs: the figures on the top row illustrate examples of the cortex of APP/PS1 mice injected with (**D**) saline and (**E**) D-Ala^2^GIP, immunohistologically stained against β-amyloid (scale bar: 880 μm). The figures in the middle row illustrate example of cortex of APP/PS1 injected with (**F**) saline and (**G**) D-Ala^2^GIP, stained with Congo red (scale bar: 880 μm). The figures on the bottom row illustrate example of cortex of APP/PS1 mice injected with (**H**) saline and (**I**) D-Ala^2^GIP, immunohistologically stained against Iba1 (scale bar: 80 μm).

There was no difference between APP/PS1 mice injected with saline or D-Ala^2^GIP in the number of plaques in the cortex (*t *= 0.21, *P *> 0.05, Student's unpaired *t-*test). Additionally, D-Ala^2^GIP peptides showed no effect on the amount of stained area for Congo red (*t *= 1.30, *P *> 0.05). However, a slight decrease in activated microglia was found in the cortex of APP/PS1 mice treated with D-Ala^2^GIP compared to the saline group (*t *= 2.00, *P *< 0.05) (Figure 10a).

#### Analysis at 12 months

There was a significant decrease in the number of plaques (*t *= 2.41, *P *< 0.01) and a lower amount of Congo red stained area (*t *= 1.96, *P *> 0.05, Student's unpaired *t-*test) in the cortex of 12 month-old APP/PS1 mice that were chronically treated with D-Ala^2^GIP. Moreover, a marked decreased in the inflammation response shown in activated microglia was found in the cortex of D-Ala^2^GIP-treated mice (*t *= 3.89, *P *< 0.001) (Figure 10b).

## Discussion

The current study provides for the first time evidence that chronic administration of D-Ala^2^GIP can improve cognitive function in WT mice and prevent deficits of learning and memory in APP/PS1 transgenic mice. Body weight, blood glucose and plasma insulin levels were monitored to demonstrate that this drug that had originally had been developed as a treatment for type 2 diabetes does not affect these parameters. Changes in blood glucose and insulin levels would be undesirable and can lead to a deficit in cognitive performance [44,51,52]. APP/PS1 mice had higher body weight at 6 and 12 months old compared to their WT age-matched littermates. At 12 months of age, all mice had a decrease in body weight, which could be the effect of regular exercise during behavioural tasks, increasing energy expenditure and reducing body weight. Higher glucose levels were also found in the APP/PS1 mice at 6 and 12 months old when compared with the WT mice. However, blood glucose levels stayed in a normal range (between 4 and 8 mM/l). Importantly, D-Ala^2^GIP treatment did not affect body weight, blood glucose levels and plasma insulin levels in either APP/PS1 or WT mice at either of the ages tested. Another important parameter to evaluate was the effect of D-Ala^2^GIP treatment on the spontaneous behaviour of the transgenic mouse, as a disturbance in locomotor activity, speed or anxiety levels could affect the learning and memory in some behavioural tasks. D-Ala^2^GIP treatment did not alter locomotor activity measured as path length and number of lines crossed, exploratory levels estimated by the number of rearing events and speed in both transgenic and APP/PS1 mice in all different ages tested. Anxiety levels were assessed by the number of grooming events and the time spent in the centre of the open-field test vs the periphery. Treatment with D-Ala^2^GIP peptides did not affect the anxiety level of mice. Moreover, spontaneous behaviour was not modified in the open-field task. The same results were found for speed during the acquisition trials in the MWM task. There was also no effect on spontaneous behaviour found previously in C57Bl/6J mice chronically injected with 25 nmol/kg D-Ala^2^GIP for at least 21 days [43].

In the ORT, mice spent more time exploring a novel object than a previously explored object if the memory of the familiar object was still intact [53,54]. Our results show that object recognition memory was intact in 6-month-old WT and APP/PS1 mice. At 12 months of age, saline-treated APP/PS1 mice failed to investigate the novel object in the ORT, and treatment with the D-Ala^2^GIP peptide rescued the memory deficit. The WT group showed intact recognition memory when tested at 12 months. In the water maze task at the age of 6 months, all mice learned the task, also in a reversal task where the platform was moved to another location. This task requires re-learning of the location and repression of the previous memory. This task is often harder to learn for animals that are impaired in learning [55]. At 12 months of age, all mice learned the acquisition and reversal task, and the saline-treated APP/PS1 group performed similar to WT mice injected with saline during the acquisition task. Importantly, both the WT and APP/PS1 group injected with D-Ala^2^GIP learned the location of the hidden escape platform faster than the saline groups. However, APP/PS1 mice displayed impaired spatial reference memory during the probe trial and reversal probe trial, as mice spent an equal amount of time in each quadrant of the pool. It seems that the 12 month-old APP/PS1 group was still able to acquire the task, but was unable to memorise and recall the exact spatial location using distal cues as the WT group did. Furthermore, WT control mice exhibited a deficit in spatial memory during the reversal probe trial. This decreased selectivity for the target quadrant in aged WT mice shows impairment in re-learning the new location of the platform. However, all mice treated with D-Ala^2^GIP showed an intact spatial memory in both recall tasks for the APP/PS1 and WT group, demonstrating the effectiveness of this drug.

Synaptic degeneration has been found in the brains of AD patients, which may be the underlying cause of memory impairment [56,57]. Thus, we examined the effect of chronic treatment of D-Ala^2^GIP on levels of the synaptic marker synaptophysin in the different layers of the hippocampus and the cortex of APP/PS1 and WT mice. Synaptic numbers in the brains of APP/PS1 mice were reduced. At 6 months, levels of synaptophysin were lower in the stratum pyramidale and polymorphic layer of the DG of APP/PS1 mice. A general decrease in synapse numbers was found in the cortex and in most of the layers of the DG in APP/PS1 mice at 12 months. This loss can explain the cognitive impairment of APP/PS1 observed during the ORT and the water maze task. Importantly, the synaptic degeneration was rescued by the D-Ala^2^GIP treatment. The 6-month-old APP/PS1 group treated with D-Ala^2^GIP displayed similar levels of synaptophysin in the polymorphic layer of the DG as in the WT group control. The deterioration of synapses in the interior cortex and in some layers of the hipppocampus of APP/PS1 was also prevented by administration of D-Ala^2^GIP in the 12-month-old group. Moreover, D-Ala^2^GIP increased the number of synapses in the cortex and the molecular layer of the DG of APP/PS1 mice at 6 months. Thus, the synaptic increase in the brains of WT mice treated with D-Ala^2^GIP could have led to the cognitive improvement. Moreover, as the reduction in the number of synapses was lower in the D-Ala^2^GIP-treated APP/PS1 mice, D-Ala^2^GIP protects synapses and rescues cognitive deficits found in the APP/PS1 mice.

We have previously shown that GIPRs play an important role in synaptic plasticity, as LTP was obliterated in the GIPR knockout (KO) mice [47], and activation of GIPRs in the hippocampus by the GIP agonist N-AcGIP enhanced the induction of LTP [38]. Thus, we assessed the effect of D-Ala^2^GIP on synaptic function of APP/PS1 and WT littermates. In this study, different aspects of *in vivo *synaptic function were evaluated, such as basic neurotransmission, LTP and gamma amino butyric acid (GABA)-ergic inhibition in area CA1 using PPF. At the ages that have been investigated, comparison of post-HFS slopes of fEPSPs revealed an increase in the D-Ala^2^GIP treatment group. Thus, chronic administration of D-Ala^2^GIP facilitated LTP induction and expression in APP/PS1 mice at 6 and 12 months of age, as well as their WT age-matched littermates. The LTP facilitation in the D-Ala^2^GIP-treatment group could be the result of an enhancement in the post-synaptic LTP induction mechanism, an increase of presynaptic transmitter release, or a change in local inhibition in the CA1 region of the hippocampus. In addition, an increased number of synapses can be the reason for enhanced LTP. The synaptophysin study showed that D-Ala^2^GIP increased the number of synapses, either by protecting them from elimination or by direct synaptogenesis. Pre-synaptic functions and inter-neuronal activity were assessed by PPF measurements. The PPF induced at short inter-stimulus intervals is considered to be triggered by pre-synaptic transmitter release facilitating processes [58], while later PPF is considered to be linked to GABA_A _and GABA_B _inter-neuronal synaptic transmission [59-61]. Even though an overall increase in PPF was found for the APP/PS1 group injected with D-Ala^2^GIP at 6 months of age, no difference in the specific inter-stimulus interval parameters has been shown. Thus, D-Ala^2^GIP does not increase vesicle release and does not seem to change the local inhibition in the CA1 region of the hippocampus, suggesting that synaptogenesis is the main factor in the enhancement of LTP.

The effects of D-Ala^2^GIP on the classic hallmarks of AD were investigated in the cortex of APP/PS1 mice. Treatment of D-Ala^2^GIP reduced the number of beta-amyloid plaques in APP/PS1 mice at 12 months of age. Moreover, the overall number of dense-core Congo red-positive plaques was reduced at 12 months. Clearly, the removal of plaques in the brain is a very desirable result, as aggregated amyloid can trigger an inflammatory response [62]. Increasingly, the role of amyloid oligomers has been brought into the focus of research. There is evidence to suggest that such soluble oligomers have neurotoxic effects [63]. It would be of interest to analyse levels of different species of oligomers in the brains of drug-treated APP/PS1 mice.

Importantly, D-Ala^2^GIP-treatment decreased the level of the activated microglia marker Iba1 in the cortex of APP/PS1 mice, showing that the neuroinflammation response was decreased by D-Ala^2^GIP-treatment. Chronic inflammation is one of the hallmarks of AD [64]. Abnormal production of pro-inflammatory cytokines by activated microglia can interrupt nerve terminal activity, leading to synapse dysfunction and loss, which correlates with cognitive decline [65-67]. Thus, the protective effect of D-Ala^2^GIP on synapses could derive from a decrease of neuroinflammation in the brain of APP/PS1 mice.

It has been shown that GIP induces progenitor cell proliferation [35] and in contrast, the lack of GIPRs in KO mice affects cell proliferation in the hippocampus [47]. In this study, chronic administration of D-Ala^2^GIP significantly increased the number of BrdU-positive cells in the DG of WT mice at 6 months of age. This observation that DAla^2^GIP enhanced neuronal progenitor proliferation in the brain, which has been previously found [35], is also of great importance, as the increase of such cells could be of use for repairing neuronal damage [68]. However, this beneficial effect was not observed in the APP/PS1 mice at 12 months old. We also tested the number of immature neurons as identified by DCX, a cell marker for immature neurons. D-Ala^2^GIP did not affect the number of DCX-positive cells in the DG of APP/PS1 mice or their WT littermates. Thus, D-Ala^2^GIP does not seem to play a role in neuronal cell differentiation. This finding confirms the previous finding that a lack of the GIPR did not affect the level of immature neurons in the DG of GIPR KO mice [47].

The data presented here have been extended in a separate study on the effects of D-Ala^2^GIP on 19-month-old APP/PS1 mice. It was found that in the very old groups, D-Ala^2^GIP still had some protective effects and reduced the plaque load, the inflammatory response (Iba1 stain) and also protected LTP from degeneration [69]. This shows that the drug has some beneficial effects even in the advanced stages of degeneration. In an additional study of the brains of these 6-, 12- or 19-month-old mice, it was found that D-Ala^2^GIP treated APP/PS1 mice had much reduced oxidative stress (8-oxoguanine levels) and astrogliosis in the brain [70].

## Conclusions

In conclusion, the results of chronic administration of D-Ala^2^GIP demonstrate that GIP analogues have a range of properties that may be beneficial in treating neurodegenerative conditions such as AD. It our study, D-Ala^2^GIP induced synaptogenesis and protected synapses, which led to cognitive improvement in WT mice and prevented memory decline in APP/PS1 mice. It has been found that GIP also promotes axonal growth after nerve crush [40]. This growth factor-like property can be of use in neuroprotection and neuroregeneration in AD. Moreover, D-Ala^2^GIP facilitated LTP, highlighting the important role of the GIPR in synaptic plasticity and confirming that synapses were not only protected from degradation in the APP/PS1 mice, but were fully functional. Importantly, D-Ala^2^GIP decreased the number of amyloid plaques and neuroinflammation in the cortex of transgenic mice. This is of great interest, as neurotoxic effects associated with plaques and inflammation are considered one of the important underlying mechanisms of neurodegeneration found in AD [71,72]. The beneficial actions of GIP suggest that the use of long-lasting analogues may be an attractive therapeutic approach for the treatment of neurodegenerative diseases such as AD. One possible mechanism of action is the re-sensitisation of insulin signaling, or the compensation of insulin de-sensitisation by GIPR activation. Further research in mouse models of neurodegenerative diseases will be required to understand mechanisms that underlie the neuroprotective properties of GIP analogues.

## Abbreviations

AD: Alzheimer's disease; ANOVA: analysis of variance; DCC: Dextran-coated charcoal; DCX: doublecortin; DG: dentate gyrus; fEPSPL: field excitatory post-synaptic potential; GABA: gamma amino butyric acid; GIP: glucose-dependent insulinotropic polypeptide; GIPR: GIP receptor; HFS: high-frequency stimulation; HPLC: high performance liquid chromatography; Iba1: ionized calcium binding adaptor molecule 1; ip: intraperitoneally; IR: insulin receptor; KO: knockout; LTP: long-term potentiation; MALDI-TOF: matrix-assisted laser desorption/ionisation time of flight; MWM: Morris water maze; NFT: neurofibrillary tangles; OLT: object location test; ORT: object recognition task; PBS: phosphate-buffered saline; PCR: polymerase chain reaction; PPF: paired-pulse facilitation; RI: recognition index; RIA: radioimmunoassay; WT: wild type.

## Competing interests

The work was funded by a grant from the Alzheimer Research UK charity. Dr Holscher is a named inventor of a patent owned by Ulster University that covers the use of GIP analogues for the treatment of neurodegenerative diseases.

## Authors' contributions

EF was a post-graduate student who conducted the research. CH was the supervisor who obtained the funding for the research and wrote the manuscript. Both authors have read and approved the manuscript for publication.
